# Aiming at a Moving Target: Theoretical and Methodological Considerations in the Study of Intraindividual Goal Conflict between Personal Goals

**DOI:** 10.3389/fpsyg.2017.02011

**Published:** 2017-11-16

**Authors:** Julia Gorges, Axel Grund

**Affiliations:** Department of Psychology, Faculty of Psychology and Sports Science, Bielefeld University, Bielefeld, Germany

**Keywords:** goal conflict, action goal, motivational interference, motivation, self-regulation, personal goals

## Abstract

Multiple-goal pursuit and conflict between personal life-defining goals can be considered part of everyday business in most individuals' lives. Given the potentially detrimental effects of goal conflict—for example, impaired well-being or poor performance—the literature on goal conflict is surprisingly scattered due to heterogeneous methodological approaches and technical terms. Little empirical research has addressed the conceptualization of goal conflict against the background of differing understandings from a structure-like and a process-like perspective. In the present article, we outline theoretical foundations of goal conflict from two perspectives: a structure- and a process-like perspective. Based on a comparative analysis and integration of these two perspectives, we systematically review empirical studies on goal conflict over 30 years of research. In doing so, we identify and discuss important conceptual dimensions of goal conflict, namely, goal conflict as a cognitive construct and an experiential instance, a focus on goal interrelations or on specific goal properties, and resource vs. inherent conflict, and the potential of these distinctions to further research on goal conflict. Finally, we present major challenges and pose questions that need to be addressed by future research.

## Goal conflict—our constant companion

When individuals are asked to list the goals they are currently striving to attain, they typically list between 10 and 15 goals (Little, 1983). Such so-called personal goals typically refer to idiographic goals of mid-level abstraction that share a life defining and rather long-term character. Although individuals may not try to pursue all of these personal goals (Riediger and Freund, 2004) simultaneously, multiple-goal pursuit can be considered the rule rather than the exception (Atkinson and Birch, 1970; Hofer, 2007; Unsworth et al., 2014). To manage multiple-goal pursuit successfully, people constantly have to check their priorities, balance their resource allocations, and plan their actions. Even so, multiple-goal pursuit may still be difficult or even impossible to achieve—especially when goals are interfering (as opposed to facilitating; Riediger and Freund, 2004) with each other. Researchers and laypeople alike speak of goal conflict when “a goal that a person wishes to accomplish interferes with the attainment of at least one other goal that the individual simultaneously wishes to accomplish” (Emmons et al., 1993, p. 531). Such goal conflict may be further differentiated according to its nature being based on either limited resources, such as time, energy, and money (*resource goal conflict*), or incompatible goal attainment strategies or end states (*inherent goal conflict*; Michalak et al., 2004; Riediger and Freund, 2004; Segerstrom and Solberg Nes, 2006).

In general, goal conflict appears to have negative effects on individuals' performance (e.g., Locke et al., 1994; Slocum et al., 2002) and well-being (e.g., Emmons, 1986; Emmons and King, 1988; Perring et al., 1988; Brunstein, 1993; cf. Gray et al., 2017). Therefore, psychological researchers from various psychological subdisciplines aim at understanding goal conflict (e.g., organizational psychology; Suliman and Abdulla, 2005; clinical psychology; Karoly and Ruehlman, 1996; or health psychology; Gebhardt and Maes, 1998). Nevertheless, the psychological literature on goal conflict is relatively scarce and rather disconnected. Major lines of research have taken different perspectives, used different wording, employed different operationalizations, and rarely referred to one another. Such lack of coherence and—eventually—conceptual clarity may threaten the validity of empirical results and lead to meta-analytic reviews being limited by a lack of comparable research designs (Gray et al., 2017). In addition, much attention has been paid to (negative) outcomes associated with goal conflict, whereas goal conflict itself—its conceptualization and delineation from its antecedents and immediate consequences on experience and behavior—have hardly been addressed.

Against this background, the present paper aims at identifying and reconciling major perspectives of research on conflict between accessible personal goals that are pursued through consciously regulated actions. In particular, we apply two perspectives to do so: a structure-like perspective that focuses on relationships between goals and their connection to central aspects of the self, and a process-like perspective that focuses on different action phases, from intention formation to goal disengagement. Based on a comparative analysis and a systematic literature review, we argue that research from these perspectives—each in its own right—has contributed substantially to our knowledge of goal conflict. However, we also outline the need to integrate both perspectives to advance research on goal conflict by considering relations and potential conflicts between goal systems across all levels of abstraction. In addition, we identify key decisions in empirical research beyond taking on one perspective or the other. Overall, we aim to present critical considerations that are necessary to define clearly the conceptualization of goal conflict underlying the respective research and to ensure that goal conflict is actually present in empirical investigations, which, in turn, is necessary to develop and test theory regarding the antecedents, mechanisms, and immediate consequences of goal conflict.

## Perspectives on goal conflicts

### A structure-like perspective

**Figure 1:**
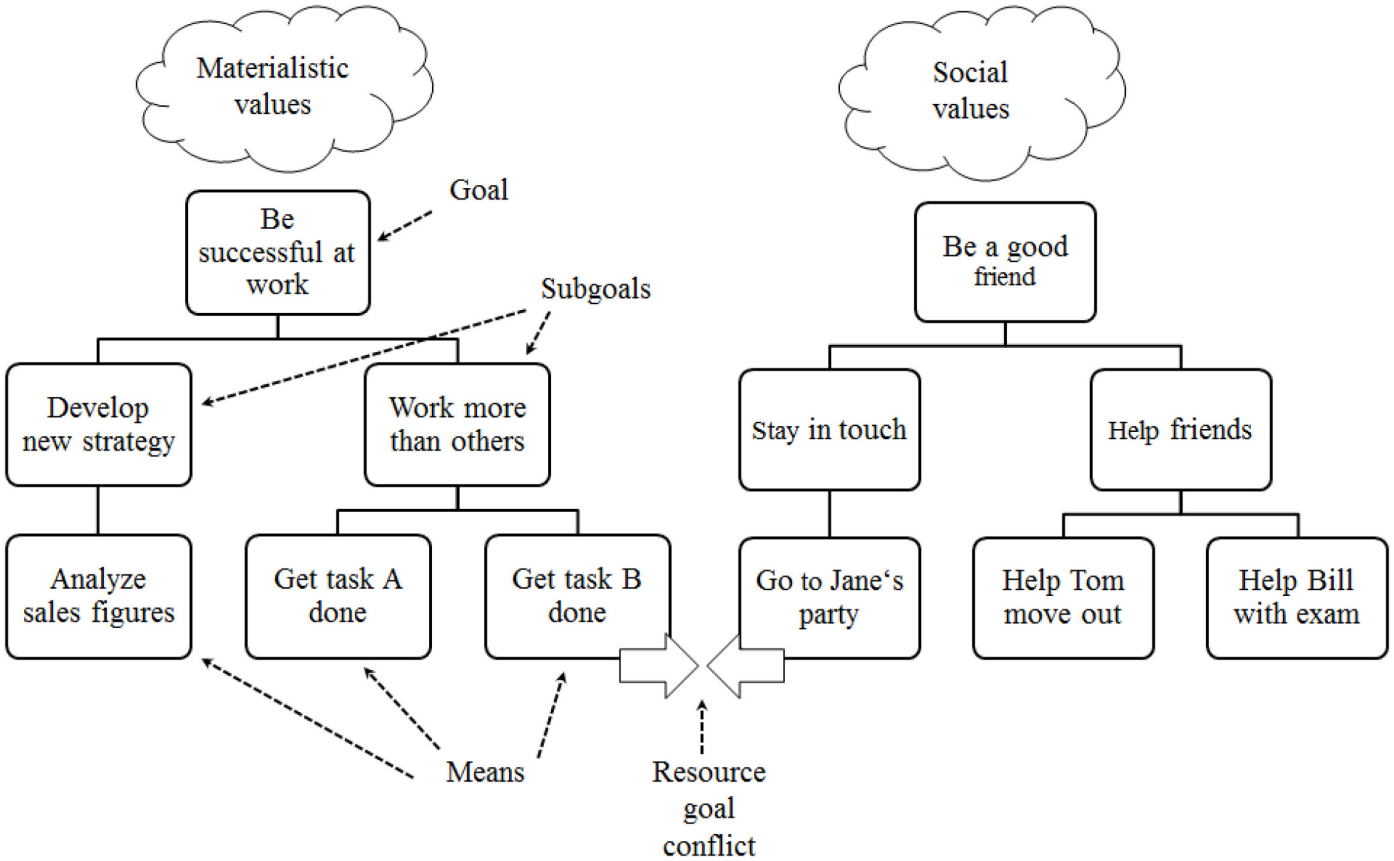
Structure of goals within goal systems [example: work vs. friends].

Goals direct rather than energize behavior (Elliot et al., 2002). They can be defined as “internal representations of desired end-states” (Austin and Vancouver, 1996, p. 338) that are structured in terms of means–end relations prototypically depicted in terms of a pyramid (see Figure 1, each side represents a goal system; e.g., Sheldon and Kasser, 1995; Carver and Scheier, 2000; Kruglanski et al., 2002). For example, in order to reach the higher-order goal to have a career, subgoals may include (a) demonstrating the ability to handle a high workload and (b) developing a new business strategy. Means to these subgoal ends could then be (a) complete task A, B, and C and (b) analyze sales figures.

Maybe the most significant continuum on which end states can be organized is generality vs. specificity (e.g., Fries et al., 2005; Lord et al., 2010). The most abstract level adheres to general values, such as self-direction and security (e.g., Schwartz, 1992). These values cannot be reached in the sense of an end state, but provide guidelines for individuals' goal setting (Fries et al., 2007). By contrast, on the most specific level, end states refer to specific tasks or actions that individuals do to accomplish their goals (down to the point of motor control; Lord et al., 2010). These tasks and actions are referred to as means people act out to attain a goal (Carver and Scheier, 1998). In between these two extremes, different levels of abstraction have been used as paradigms to study goal-directed behavior guided by personal goals (e.g., possible selves; Markus and Nurius, 1986; aspirations; Sheldon and Kasser, 1995; life tasks; Cantor et al., 1986; personal projects; Little, 1983; personal strivings; Emmons, 1986; current concerns; Klinger, 1987; personal goals; Riediger and Freund, 2004). In the present contribution, we focus on these personal goals defined as idiographic, mid-level goals that direct people's course of life over a longer period.

With the highest-order goal at the apex of the goal system, all subgoals are supposed to serve to attain or at least approach the highest-order goal. As long as the highest-order goals do not contradict each other, a person's goal systems should form a coherent structure. However, sometimes people pursue goals that are not derived from the highest-order goals, for example, because they adopt goals from other people. These goals may run counter to a person's self-chosen personal goals (Sheldon and Elliot, 1998) and may not be in accordance with the self's core values, plans, and self-defining aspects (Sheldon and Kasser, 1995).

Making things even more complicated, pursuing exclusively self-generated goals does not guarantee that all of these goals are truly “personal.” According to Sheldon and Elliot (1998), self-generated goals “can feel just as authoritarian as external rules and constraints” (p. 546), when the reasons behind these goals express a more externally controlled form of motivational regulation (Ryan and Deci, 2000). Thus, the structure of individuals' goals is highly complex and hold the potential for contradictions and friction between the goals.

Building on the concept of hierarchically structured goal systems, Sheldon and Kasser (1995) distinguished two kinds of coherences within and between goal systems, which, if not fulfilled, holds the potential for goal conflict: Vertical coherence occurs when subordinate goals are instrumental for the attainment of superordinate goals. Accordingly, vertical interference refers to situations in which individuals pursue goals that are not, or not well, connected to any self-defining values. By contrast, horizontal coherence describes facilitative relations between goals that typically belong to different goal systems. Thus, horizontal interference typically refers to instances of goal conflict between goals on comparable levels of abstraction, which will take center stage in our review.

### A process-like perspective

Having set a personal goal is not necessarily equivalent to pursuing this goal. In many cases, there may be a substantial time lag between goal setting, goal implementation, and successful goal attainment. In order to understand the difficulties of multiple-goal pursuit, it is useful to examine the various sequential steps goal-directed behavior typically follows and outline potential for goal conflicts along the way.

**Figure 2:**
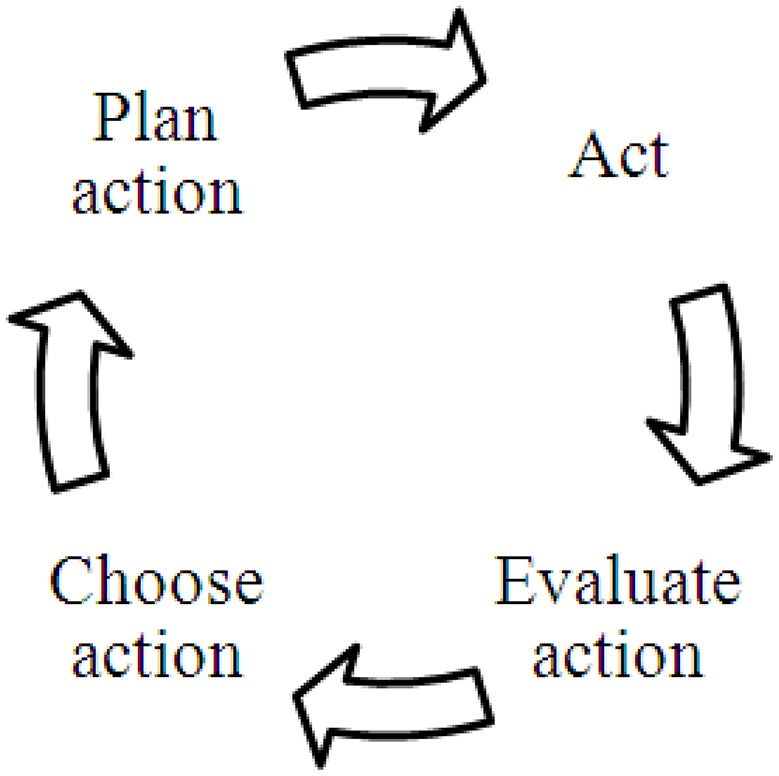
Process model of goal-directed action [example: single-goal perspective]. This figure merges the linear model of action phases (Gollwitzer, 1990; Heckhausen, 1991) and cyclical models of self-regulation (e.g., Zimmerman, 2008) to reflect the ongoing stream of individuals' actions.

According to the Rubicon model of action phases (Gollwitzer, 1990; Heckhausen, 1991), goal-directed behavior breaks down into four action phases and three transition steps, depicting the prototypical process of single-goal pursuit within a self-regulation cycle (cf. Boekaerts et al., 2000; Vohs and Baumeister, 2004; Zimmerman, 2008, see Figure 2).

In the first, pre-decisional phase, individuals choose which of their several possible goals they prefer to pursue (*choose action*). Decisions such as these are considered to follow the mechanism of modern expectancy-value theories (e.g., Wigfield and Eccles, 2000). The more likely the successful attainment of a possible goal and the higher the value attributed to it, the higher its preference. The pre-decisional phase is the one in which the notion of conflicting goals becomes apparent. Here, the competition between one's several personal goals takes place when, for example, school and leisure goals are simultaneously activated (Fries et al., 2008; Grund et al., 2014) or work-related goals compete with private life goals (Eby et al., 2005).

A deliberate goal intention in terms of a feeling of determination or commitment with regard to the desired end state characterizes the transition to the second, pre-actional phase (*plan action*). Once the choice to realize a particular action is made, one has crossed the Rubicon (i.e., literally, there is no way back). In the planning phase, goal-related thoughts and feelings are directly related to action (i.e., identify strategies to attain a goal, choose means). The more specific these plans, the higher the likelihood of successful goal attainment (Gollwitzer, 1999). In planning goal pursuit, limited resources or incompatible goal attainment strategies may become salient if the individual takes competing means attached to different goal system into account.

The next transition is characterized by the initiation or onset of action (*act*). Hence, in phase three, individuals act according to their plans and try to reduce the discrepancy between the current status quo and the desired end state. According to the Rubicon model, the clear-cut decision for one action goal (presumably the higher valued, more feasible alternative at a given moment), the inception of a focal goal intention, and the devaluation of alternative goals, enables people to pursue one goal without being affected by (potentially conflicting) alternative goals (Gollwitzer, 1990; Shah et al., 2002; Fishbach et al., 2010; Unsworth et al., 2014). However, recent research on motivational interference has demonstrated that goal conflict may pose difficulties for people attempting to stick to their plans, or may distract them and cause them to doubt even after having decided on a focal goal (Fries et al., 2008; Grund et al., 2015). Research on motivational interference has revealed negative effects of conflicting goals on individuals' experience and performance, even when the dismissed alternatives are only mentally present and no actual switching has occurred (Fries et al., 2008).

The last transition refers to the achievement of the goal. After a person has reached a designated goal, s/he evaluates in the last, post-actional phase, whether the actual outcome state matches the desired and expected outcome state (*evaluate action*). Here, the competition between several goals may become vital again. Thus, when individuals evaluate their actions, they may realize that they have sacrificed one goal in favor of another without intending to do so, which may have a range of cognitive, affective, and/or behavioral consequences. For example, people may regret their actions or delve in counterfactual thinking (Gilovich and Medvec, 1995; Kuhnle and Sinclair, 2011). In addition, (potential) failure to attain (or approach) one of the goals should shift back to higher-level goals, and people may have to disengage from or revise a goal, or draw consequences for future action (Kehr, 2003). Future pre-decisional and pre-actional considerations may be derived out of the concluded goal-pursuit attempt.

### Reconciling both perspectives into an integrative framework for thought

Our key proposition is that conceptualizations of goal conflict need to consider both a structure-like and a process-like perspective simultaneously. Hence, drawing on higher-order (personal) goals needs to consider necessary subgoals and means from the process of goal pursuit to identify goal conflict. In turn, drawing on competing means during the process of goal pursuit needs to consider the anchoring of these means in superordinate subgoals of the respective personal goal and/or the personal goal itself. Considering both perspectives challenges the implicit assumption that goal conflict manifests itself primarily as same-level conflict.

To integrate the two perspectives outlined above, we will theoretically trace the path of goal conflict through levels of abstraction and action phases. The potential for goal conflict is set up within the higher-order goal structures, when one has too many goals that are drawing on limited resources, or when one sets goals that are attached to incompatible goal attainment strategies or end states. At this stage, if individuals are already planning ahead across levels of abstraction, they may become aware that they do not have the resources to pursue either goal, or that the end states are incompatible. Therefore, they may feel blocked because they do not know what to do, or commitment for each goal may be reduced.

After choosing a goal to pursue, this goal will be activated and translated into means to act out. At this level, goal conflict likely becomes salient in individuals' minds because individuals actually have to deal with competing means and goals when attempting to move forward with their action. If individuals choose one particular goal to pursue and continue with their actions, they may still experience motivational interference. Depending on one's decisions and actions, goal conflict may affect higher levels of the relevant goal systems during and/or after goal pursuit if individuals reflect on the implications of their actions for their personal goals. For example, realizing that both goals cannot be attained, individuals may have to disengage from or change a goal. In each phase, individuals may refer back to higher levels of their goals to assure themselves of the goals' relevance and/or to try to find a solution.

Thus, goal conflict detected in the structure of a person's goals may, but does not necessarily, affect his or her actions (i.e., become *action-relevant*). That is to say, individuals may carry with them goal conflict without being bothered. As long as individuals do not actually try to pursue both (all) conflicting goals at the same time, they may simply not notice interfering goal interrelations. By contrast, choosing a goal and planning one's action may be heavily impaired by goal conflicts. Here, goal conflict touches the level of action, and individuals become aware of goal conflict they may not have noticed before because they have not yet tried to attain these goals.

As can be seen in Figure 1, on an abstract level, the higher-order goals of being a good friend and being successful at work do not necessarily conflict with each other. The same can be true for midlevel goals, such as to develop a new business strategy and stay in touch. However, on the specific-level of current action, a person may not have the time to pursue work-related and social goals alike. In addition, two means may essentially come into conflict even though they may be instrumental for the same midlevel goal (e.g., “going to Jane's party” and “helping Tom move” are both instrumental for “staying in touch with friends”). These instances are likely to represent action-relevant resource goal conflict due to a lack of time, money, or energy. Similarly, inherent conflict probably manifests itself on the specific-level of a self-regulation hierarchy and becomes action-relevant because opposing standards for self-regulation most likely become operant with specific means-end relations. For example, a manager may try to be friends with his or her staff while at the same time evaluate their performance.

In sum, (antecedents of) goal conflict established on higher levels of abstraction may impact individuals' actions and performance on the level of means, whereas (consequences of) goal conflict on the level of means may feedback to higher levels of abstraction and impair individuals' well-being and actions in the future. A structure-like and a process-like perspective (and research) on goal conflict thus differ by the consideration vs. exclusion of the level of abstraction involved in the conflict under investigation. Therefore, an integrated perspective is better suited to establish a full account of the conceptualization of goal conflicts, which may be located on and across all levels of abstractions and action phases.

### When is a goal conflict a goal conflict? answers from an integrative perspective on goal conflict

Returning to the definition of goal conflict cited in the introduction—“a goal that a person wishes to accomplish interferes with the attainment of at least one other goal that the individual simultaneously wishes to accomplish” (Emmons et al., 1993, p. 531)—we may say that this definition is valid from both perspectives. Attempting to translate this definition into a watertight operationalization to measure personal goal conflict from one perspective or the other is, however, challenging, and may differ depending on the perspective we take.

First, looking at goal conflict through structure-like glasses calls for an analytic approach. Goal structures would need to be analyzed against the background of the individual's resources to evaluate whether the goal systems are consistent within themselves, consistent across one another, and attainable given the resources available. Thus, taking on a structure-like perspective in empirical research would need access to individuals' goal structures by, for example, asking them (but see Kruglanski et al., 2002, for an experimental approach).

From a process-like perspective, the definition requires an *action-relevant* goal conflict that is noticeable at least in a person's mind if taken literally (i.e., “interferes” as present tense, for example, by sorting through conflicting goals to choose one goal that enters the planning phase, by switching tasks or by experiencing motivational interference). Unfortunately, action-relevance is hardly defined in experiential terms, which makes it difficult to pinpoint action-relevant goal conflict based on specific experiential instances. Action-relevance may emerge in different and more or less obvious forms in terms of interruptions of a person's course of action. For example, a rather obvious experiential instance of action-relevant goal conflict may lead a person to switch between actions, whereas a less obvious experiential instance may lead him or her to “only” shift resources from one goal to another, or to deliberately think about how he or she can reach both goals. Because individuals dispose of various strategies to deal with potentially conflicting goals, however, they have many ways to prevent a goal conflict from taking negative effect on their actions. Hence, defining what shall be considered as goal conflict and what shall not (yet) constitute goal conflict will be necessary.

To identify potential experiential instances indicating goal conflict, a discussion along the action phases may be helpful. In particular, defining which action phase a goal conflict must have reached to fulfill its definition is important. For example, rumination or difficulty to decide on a focal goal may or may not be considered as a goal conflict. One may argue against recognition of rumination as a goal conflict because the action has *not yet* started. Conversely, however, one may argue in favor of recognition as a goal conflict because rumination may hinder individuals from starting an action, thereby impairing the course of action.

During the action phase, goals may be pursued and attained in sequence—i.e., act on and attain goal A first, and then turn to goal B—so that limited resources will not be overused. More recent descriptions of the Rubicon model also allow for suspending and resuming goal-pursuit while pursuing another goal in the meantime (Kleinbeck, 2009). Thus, individuals may switch between goals in terms of their regular course of action, for example, because the active goal depends on attaining another goal first, or because the opportunity to pursue another goal is more favorable than sticking to the active goal. When individuals stick to one goal, they may still experience motivational interference (Grund et al., 2014). In addition, individuals may be able to implement multifinal means related to two or more goals they want to pursue (cf., Fishbach and Ferguson, 2007; Köpetz et al., 2011). Finally, in some cases, a rethinking of one's goals and priorities, or even disengagement from a goal (Brandstätter and Schüler, 2013), would be the logical consequence of goal conflict because goal attainment may be impossible. Hence, if an individual uses any of these strategies to pursue potentially conflicting goals and, thereby, succeeds in not having their actions impaired by the goal conflict, one could say that he or she did not have a goal conflict. However, one could also say that individuals had to spend energy on regulating their course of action to avoid (further) impairment of their course of action by these (potentially) conflicting goals, which may qualify as goal conflict. In other words, if a person anticipates goal conflict and takes precautions to prevent this goal conflict from becoming action-relevant (i.e., by changing the schedule for goal attainment), did he or she have goal conflict at all? It is arguable—and subject to definition—whether situations in which individuals may go on with their course of action through successful self-regulation or employment of multifinal means actually fulfill the definition of goal conflict. Overall, this discussion comes down to the question of what kind of interruptions qualify as indicators of goal conflict.

As is evident from these considerations, research on goal conflict needs a careful definition of goal conflict as a research objective, and an equally careful operationalization. From an integrative perspective, we would like to suggest developing different, but related, conceptualizations, for example, goal conflict in a narrow and in a broad sense. A narrow sense could focus on goal conflict that has reached the level of action, whereas a broad sense could encompass any—even small and merely mentally represented—interruptions of one's course of action.

With respect to goal conflict research, different conceptualizations of goal conflict derived from the two perspectives—roughly speaking, as a non-action-relevant goal conflict concluded from one's goal structure or as an action-relevant goal conflict indicated by a specific experiential instance—determine the processes and mechanisms that may lead to goal conflict formation (i.e., its antecedents) and explain how goal conflict affects individuals' regulatory effectiveness and well-being (i.e., its consequences). To establish a sound theoretical and empirical account of goal conflict and produce comparable study designs, some common ground would be helpful. In the following, we will therefore investigate existing empirical approaches to the study of goal conflict to see which perspective on goal conflict is prevalent in empirical studies and how existing theoretical conceptualizations and empirical approaches may be used systematically to further our understanding of goal conflict. Building on the integrated perspective, we will then discuss what needs to be taken into consideration in the design of empirical studies to illuminate antecedents and consequences of goal conflict, which constitute key questions within goal conflict research.

## Methodological approaches in existing research on goal conflict

To gain an overview of existing empirical research on goal conflict, we conducted a systematic literature review covering research on goal conflict from 1985 to 2015 in the PsycInfo database. We chose 1985 as a starting point because research on idiographic, mid-level personal goals gained increased importance at the beginning of the 1980s, producing several seminal works (e.g., Little, 1983; Emmons, 1986; Klinger, 1987). We searched for publications with the keyword “goal conflict” and limited the results to peer-reviewed academic journal articles in English with goals, conflict, and empirical study as major subjects. We found 161 articles matching these criteria. Next, we scanned all abstracts to determine whether the study addressed naturally occurring intraindividual goal conflict between two or more personal goals. Hence, we excluded studies on interpersonal goal conflict, organizational conflict, conflict between pursuit vs. disengagement regarding a single goal, cognitive conflict between incompatible tasks, and non-conscious goal conflict. Following these guidelines, 35 articles remained in our final review sample and were inspected in more detail (see references highlighted by an asterisk).

Overall, we found manifold approaches for assessing and investigating goal conflict. Some differences were quite obvious, whereas other were rather subtle. To provide a structured presentation of our essential findings, we summarize three key decisions in goal conflict research in the following chapters. In principle, the different approaches to the study of goal conflict may be combined in any way. In the literature, however, some combinations appear more often than others. In the following sections, we will examine these empirical approaches in more detail.

### Measuring goal conflict from a structure-like vs. a process-like perspective

Conceptualizing goal conflict from a structure-like vs. a process-like perspective leads to major differences in its operationalization and, thus, the interpretation of empirical findings. A structure-like perspective prototypically leads to measuring goal conflict as a cognitive construct (i.e., mental representations of goal conflict), whereas a process-like perspective would lead to measuring goal conflict as an experiential instance (i.e., recollections of a person's past or anticipations of his or her future experiences). Along this distinction, empirical approaches to capturing participants' goal conflicts may rely on abstract ratings, indicating cognitive constructs (“one goal striving *is seen* […] *as interfering* with the achievement of other strivings”; Emmons and King, 1988, p. 1,041, italics by authors), or tap participants' experience presumably related to goal conflict (“pin down people's *experiences when actually facing* a particular goal conflict”; Gorges et al., 2014, p. 478, italics by authors).

Taking on a structural perspective, many researchers ask participants to rate the extent of conflict (or interference) between two goals (Emmons and King, 1988; Sheldon, 1995; Kehr, 2003; Stangier et al., 2007; Wiese and Salmela-Aro, 2008; Kelly et al., 2011; Boudreaux and Ozer, 2013). A seminal study by Emmons and King (1988) draws on judgements of how one goal affects the attainment of another goal (“Does being successful in this striving have a helpful, a harmful, or no effect at all on the other striving?” p. 1,042) regarding several goal interrelations. Similarly, Kehr (2003) had participants report their cognitive representation of goal interrelations, asking “Does the pursuit of this goal support or inhibit the other goal, or does it have no effect on it?” (p. 199). Still using a cognitive construct approach, but framing the question a bit differently, participants in Boersma et al.'s (2006) study indicated their agreement to items such as “Pursuing this goal will be at the expense of other important goals I want to achieve” (p. 931), reflecting a conceptualization of goal conflict as a property of one (or more) goals (see section Measuring Goal Conflict Based on Goal Interrelations vs. Single-Goal Properties).

Taking a different avenue, researchers who are interested in investigating action-relevant goal conflict typically try to pinpoint a goal conflict that is marked by a particular experience, such as an interruption of one's course of action (Gorges et al., 2014), pose more or less open questions (Lee et al., 1991; Berrios et al., 2015), or refer to a specific affect presumably related to goal conflict (Suliman and Abdulla, 2005; Bailis et al., 2011). Measuring goal conflict as an experiential instance, however, is quite a challenge when the experience—and thereby the assessment of goal conflict—is meant to be unrelated to potentially negative effects of goal conflict, which oftentimes is the relation under investigation.

Methodological approaches to tapping participants' factual experiences encompass direct and indirect questioning strategies. For example, Berrios et al. (2015, p. 758) asked participants to “recall as vividly as possible a recent conflicting goals event” and provided “a specific definition of goal conflict and some examples.” By contrast, Gorges et al. (2014) drew on the concept of motivational interference as an indicator of goal conflict, asking participants “to remember a situation in which they felt torn between two activities within the last couple of weeks” (p. 478). Next, participants may be asked to report general ratings of how much goal conflict they experience (e.g., “To what extent did one goal have harmful effects on the other goal?” Berrios et al., 2015, p. 758; Kuhnle et al., 2010) and/or ratings of items that reflect experiences associated with goal conflict (Zaleski, 1987; Lee et al., 1991; Gebhardt and Maes, 1998; Slocum et al., 2002; Suliman and Abdulla, 2005; Gorges et al., 2014).

### Measuring goal conflict based on goal interrelations vs. single-goal properties

Seen from a different angle, empirical research differs in its conceptualization as a specific goal interrelation vs. a characteristic attached to the goal. Drawing on the idea of horizontal interference within goal structures, Emmons and King (1988) have introduced an idiographic-nomothetic goal conflict assessment in terms of a matrix. Assuming that goal conflict primarily occurs on lower rather than higher levels of abstraction, empirical approaches to elicit personal goals and, subsequently, goal conflict, typically focus on midlevel goal concepts, such as personal strivings (e.g., Emmons and King, 1988; Riediger, 2007). Following Emmons and King (1988), many studies take into account several goal interrelations and their potential for goal conflict. Hence, participants first list the goals they are currently striving to attain (Michalak et al., 2004). Next, using these goals as columns and lines—thereby spanning a matrix (e.g., Emmons, 1986)—participants evaluate the interrelation of each pair of goals. Goal interrelations may be supportive (i.e., attainment of goal A facilitates attainment of goal B), indicated by positive values; conflictive (i.e., attainment of goal A interferes with attainment of goal B), indicated by negative values; or neutral, indicated by zero. Finally, the sum of adding up all the values reflects participants' overall extent of goal conflict. More precisely, the final score represents the weighted sum of goal conflict counterbalanced by facilitative intergoal relations. Next, the overall extent of goal conflict (i.e., the index) is related to outcome variables at more general levels, such as affective well-being (Emmons and King, 1988; Boudreaux and Ozer, 2013), and job and family satisfaction (Wiese and Salmela-Aro, 2008). This procedure has been used widely in goal conflict research focusing on a range of goal interrelations (Sheldon, 1995; Kehr, 2003; Hardy et al., 2011; Kelly et al., 2011; Presseau et al., 2015), or on one particular goal interrelation (Locke et al., 1994; Karoly and Ruehlman, 1996; Etkin et al., 2015).

Unlike approaches based on goal interrelation, other authors focus on single goals and conceptualize goal conflict as a goal property (Cantor et al., 1992; Boersma et al., 2006; Li and Chan, 2008; Hardy et al., 2011). Thus, the assessment of goal conflict is tied to participants reporting on goal properties using items such as “How much conflict does working on this task engender in your life?” (Cantor et al., 1992, p. 646). These items may refer to the actual goal conflict presumably caused by the goal or the potential for goal conflict, respectively.

### Measuring resource vs. inherent goal conflict

Despite fundamental conceptual differences between resource goal conflict and inherent goal conflicts, which may well be relevant for goal conflict formation and consequences, most studies do not specify which type of goal conflict they ask for (e.g., Emmons and King, 1988). If they do, the type of goal conflict to be reported—i.e., resource conflicts, inherent conflicts, or both—is elicited by prescribing the goal conflict in question or by using specific instructions to trigger the specific type of goal conflict. As a prominent example of research on resource goal conflict, Locke et al. (1994) conducted a study with participants who typically have limited resources but unlimited goals to achieve (i.e., researchers having research and teaching duties). Similarly, Slocum et al. (2002) focused on sales people who face resource goal conflict due to additional sales goals. Hence, these studies imply that they focus on resource goal conflict via the setting of the study. A more explicit approach has been followed by Gorges et al. (2014), who have suggested a certain type of goal conflict in their instruction. Their participants were junior scientists who have been introduced to the problem of limited time resources (“…time resources for the realization of these numerous intentions are naturally limited. This may lead to conflicts, e.g., should I prepare my lecture today, or continue to write my research paper, or participate in the faculty assembly,” p. 478). Next, participants were asked to report a recent conflict along these lines. Hence, the instruction in this study focuses on resource goal conflict without using this particular technical term.

Contrasting numerous studies on resource goal conflicts, research on inherent goal conflict is scarce. From our sample, only five studies explicitly address inherent goal conflict as distinguished from resource goals conflict. Most studies tap inherent goal conflict by prescribing at least one goal (intelligence vs. romance goals of women; Park et al., 2011); drinking alcohol but not driving while being drunk (Liourta and van Empelen, 2008); saving vs. spending money (Loibl, 2009); stopping smoking (McKeeman and Karoly, 1991), and asking for its interrelations with other goals or the extent of its conflict with other goals in general. It is striking that many of these studies focus on at least one avoidance goal. Maybe avoidance goals many a time do not require resources, such as time or money, and, therefore, are more likely to be part of an inherent goal conflict. Nevertheless, a broad conceptualization of goal conflict would acknowledge the self-regulating energy required to avoid, for example, smoking as a resource as well (Muraven and Baumeister, 2000). Hence, avoidance goals can be part of resource goal conflict, and ceasing smoking, for example, may not be part of an inherent goal conflict after all. Only one study in our sample used the distinction between resource and inherent goal conflict on self-generated goals based on a matrix (Segerstrom and Solberg Nes, 2006), but used trained raters to establish the difference between the two types of goal conflict. Apparently, the distinction between the two types of goal conflict is quite complex, which makes it difficult to ask participants to distinguish between them.

## Lessons learned from reviewing current approaches to assess goal conflict

### Conceptualizing goal conflict as a cognitive construct vs. an experiential instance

Taking on a structure-like perspective on goal conflict and, consequently, using cognitive construal as an empirical approach to measure goal conflict goes along with certain presuppositions that should be clear to researchers and readers alike. Most importantly, the basic assumption is that goal conflict is what people report as goal conflict and, therefore, is a highly suggestible and potentially malleable construct. In fact, perceiving—and consequently reporting—goal conflict may depend on a range of personal and contextual factors. With respect to personal factors, Freitas et al. (2009) asked participants for their perceived goal conflict, but deliberately manipulated the level on which participants' elicited personal goals by a preceding task. As expected, reporting interfering goal interrelations depends on the structural level of goals: participants reported more goal conflict if they had generated personal goals from a lower structural level. Segerstrom and Solberg-Nes' (2006) work on the role of optimism represents another study challenging the validity of direct judgements of goal conflict: Independent raters evaluated conflict between personal goals reported by the participants. The extent of conflict was significantly related to participants' optimism. With respect to contextual factors, participants' current situation or recent experiences may affect their judgement of goal interrelations. For example, after a particularly stressful week at work, resource goal conflict may be more salient than usual.

The perception of goal conflict may also be influenced by the goals' properties. Goals can be described in more detail in terms of their goal properties (cf. Austin and Vancouver, 1996) and their underlying motivation (e.g., Sheldon and Elliot, 1999). As goal properties—such as temporal range, goal commitment, goal attainability, and goal self-concordance—have been shown to affect single goal processes, they likely play a critical role in multiple-goal pursuit in the case of goal conflict as well. For example, goal self-concordance has been found to affect whether people experience multiple-goal pursuit as psychological strain vs. challenge (Senécal et al., 2001; Gorges et al., 2014). Hence, feelings of interference may vary according to the extent to which goals are pursued with self-determined motivation (Senécal et al., 2003; Ratelle et al., 2005; Grund, 2013). This may lead to biased results when participants report on how many goal conflicts, or how much overall goal conflict they perceive.

One may argue that the subjectively perceived extent of goal conflict is what affects people's actions and well-being and, therefore, is a reasonable indicator of goal conflict. From this perspective, however, finding valid evidence through methodological approaches that require participants to carefully assess many of their goal interrelations and literally search for conflict within their goal structure may be difficult because such procedures will probably increase the salience of goal conflict. In addition, the goal conflict reported may mix action-relevant and non-action-relevant goal conflict. In fact, using a cognitive judgement approach likely attracts attention to goal conflict existing “only” in people's mind, which may not become action-relevant in the near future (if at all), and, thereby, may have “fake” negative effects on people's well-being. Thus, conceptualizing goal conflict as a cognitive construct and, consequently, asking participants to construct these goal conflicts for the study could result in methodological artefacts.

Taking on a process-like perspective of goal conflict, however, is anything but an easy solution to these measurement problems. Studies using experiential instances to assess goal conflict mostly rely on feelings of interference or conflict to evaluate the existence and the extent of individuals' goal conflict. Using the effect of a goal conflict on individuals' experience as an indicator of its existence obviously is problematic. If the experiential instance is intertwined with possible effects of goal conflict, disentangling existence and effect of goal conflict is difficult, if at all possible. Hence, by considering (only) negative affect as an indicator of goal conflict (e.g., rating frustration; Suliman and Abdulla, 2005; rating negative effects on other valued goals; Slocum et al., 2002), the cart is put before the horse. Measuring goal conflict based on its consequences precludes insights into how goal conflicts become effective, and blurs conceptual clarity of goal conflict, such as its consequences and, probably, its antecedents.

To delineate existence and effects of goal conflict, experiential instances of goal conflict should be carefully distinguished from potential consequences of goal conflict. The major challenge, however, is that we do not know for sure which more or less neutral experiential instances may indicate goal conflict. In an attempt to minimize intermixture of existence and effect, Gorges et al. (2014, p. 478) took recourse to the phenomenon of motivational interference when they asked individuals to “think back to the last couple of weeks and try to remember a particular situation in which you ‘felt torn’ between two intentions” in order to pinpoint one particular goal conflict that had an effect on individuals' actions. This approach may open a potential avenue for designing experiential instances in empirical research. Nevertheless, approaches based on experiential instances typically do not take into account that all goals are embedded in a complex structure of goal systems. Consequently, which goals (i.e., on which level, belonging to which goal systems) actually cause the conflict that impairs action may remain unclear.

### Focusing on goal interrelations vs. single goal properties

Following the conceptualization of goal conflict as an interfering relation between two goals (Emmons et al., 1993), idiographic-nomothetic approaches that elicit personal goals and have participants judge their interrelations come very close to theoretical assumptions. From this viewpoint, focusing on goal interrelation(s) apparently is more precise than asking for conflict in terms of a single goal property. In addition, the latter makes great demands on participants' ability to analyze and reflect their goal, its embeddedness in goal structures, and consequences of goal pursuit. Hence, goal conflict should generally be assessed based on goal interrelations rather than goal properties.

Reviewing empirical studies using the goal interrelations approach, however, revealed that many of them did not focus on a single goal conflict. Rather, they focus on a summary of participants' current or future goal conflicts, which does not seem apt to investigate antecedents and consequences of goal conflict in much detail. The major reason for this evaluation is ambiguity in assigning effects to a particular goal conflict involving specific goals. Hence, an overall goal conflict index would reveal if only one of the goal conflicts was responsible for negative effects whereas the others have neutral or even positive consequences. Because theory and empirical findings suggest that some goal conflicts may be more detrimental than others (Gorges et al., 2014), pooling goal conflict into one variable blurs the contribution of single goal conflicts to overall effects.

Another challenge regarding the methodological procedure of a goal matrix is that instructions typically aim at eliciting people's goals from only one level, but cannot ensure that all reported goals actually are on the same level of abstraction. In fact, the matrix approach would not detect if all facilitative goals belonged to one superordinate goal. Hence, both the definition of goal conflict as the pursuit of one goal impairing attainment of the other goal, and its operationalization, appears rather unspecific. In addition, the potential inducement of negative effects by the methodology and the susceptibility to bias—not least because of moderators such as personal dispositions or goal properties—constitute serious threats to the validity of empirical results. Nevertheless, positive aspects of the matrix approach include its pioneering contributions to research on goal conflict and its ability to tap individuals' diverse, idiographic goals and goal interrelations.

### Resource goal conflict vs. inherent goal conflict

The key decision resource goal conflict vs. inherent goal conflict seems closely related to theoretical underpinnings of a study. Our review revealed a preponderance of studies on resource goal conflict, which is, however, mostly implicit. Given the sheer number of goals individuals pursue and the natural tendency to form a coherent goal structure (Little, 1983), the literature may reflect the stronger prevalence of resource goal conflict compared to inherent goal conflicts. Nevertheless, assuming that inherent goal conflict occurs when individuals take on goals set by other individuals (or goals that are at least shaped by expectations of significant others) —e.g., at work, within families—this type of goal conflict should be very relevant for individuals' lives as well. Hence, investigating inherent goal conflict in its own right and comparing antecedents, experiential instances, and consequences to resource goal conflict is an important yet insufficiently engaged task.

Contexts in which both resource and inherent goal conflict occur may be particularly fruitful to the study of inherent goal conflict. In Germany, for example, teachers' workplace is considered as one which generates resource goal conflict and inherent goal conflicts. More specifically, teachers typically have to attend to various tasks prior to, during, and after classes (cf. Grund et al., 2016). In addition, teachers pursue fundamental professional goals that are incompatible with one another, known as antinomies (e.g., promoting autonomous actions in a heteronomous environment; Helsper, 1996). Initial steps toward assessing resource goal conflict and inherent goal conflict, each in its own right, have recently been taken by Neumann et al. (unpublished manuscript).

Even though we know little about antecedents of goal conflict, we may assume that resource goal conflict primarily challenges individuals' self-regulation competence. By contrast, it may not constitute a primarily logistic problem of resource allocation. Instead, inherent conflict refers to intrapsychic frictions in the sense of self-discordance (cf. Sheldon and Kasser, 1995), and, therefore, may be especially pivotal for psychological well-being. Hence, we may assume that both antecedents and consequences differ depending on the type of goal conflict. The type of goal conflict may be a moderator, or the types may even constitute distinct theoretical constructs. Either way, researchers need to address both types of goal conflict in their own right.

### Further options in the study of goal conflict

Beyond the three key decisions, we identified a number of further options researchers can choose when studying goal conflict. As a subtle but still important difference, empirical studies on goal conflict vary in the tense used in their instructions. The abovementioned studies on goal interrelations typically ask participants to list personal goals they are *currently* striving to attain and to rate the extent of goal conflict (Emmons and King, 1988). By contrast, some researchers used past tense wording to elicit goals (or activities) that participants *have been* trying to attain in the recent past and for which they rate how much these goals have been conflicting (Perring et al., 1988; McKeeman and Karoly, 1991). Although, this procedure does not instruct participants to deliberately focus on goal conflict that has affected their actions, referring to the past may be more likely to tap action-relevant goal conflict.

Beyond that, some authors were interested in very specific conflicts involving one particular goal. In such cases, the goal of interest—for example, physical exercise—has been prescribed during the study and related to a second self-reported personal goal (e.g., Carraro and Gaudreau, 2015). Taking a different approach, other studies lack a direct assessment of (the existence or extent of) goal conflict. Instead, these studies rely on the existence of goal conflict solely inferred from contextual factors such as being in a specific situation (e.g., reporting strong physical exercise and academic goals, Bailis et al., 2011; change of residence; Segerstrom, 2001, 2006) or reporting certain characteristics theoretically associated with goal conflict (e.g., obsessive passion; Bélanger et al., 2013; see also Loibl, 2009; Park et al., 2011). We consider these requirements regarding the presence of goal conflict rather lax.

## Recommendations for future research

### Theoretical implications: two requirements of integrating perspectives on personal goal conflict

The complexity of goal conflict requires a careful definition of goal conflict as a research objective, and a close look at research findings, to figure out which aspect of goal conflict has actually been addressed in the literature or will be addressed in the respective study. Integrating a structure-like and a process-like perspective should foremost be done by outlining an integrated conceptualization of goal conflict as a basis for empirical research addressing goal conflict. In particular, we need to address conflicting goal interrelations across levels of abstraction that fuse a structure-like and a process-like perspective. For example, from a structure-like perspective, goal conflict may arise when people set goals that are not in line with the goals they already have. Considering the process-like perspective as well, we need to ask to what extent people (would need to) anticipate means to attain their goals, foresee their future goal-directed behaviors, and predict their resources, to (be able to) decide whether a new goal will cause conflict. In this regard, personal characteristics such as ability to and motivation for complex and reflective thinking may play an important role.

Many studies taking on a process-like perspective have linked the experience of personal goal conflict to behavioral and/or experiential consequences, whereas they mostly lack the link between processes on an action-level to processes located on higher levels of abstraction that occur before or after taking action. Thus, research investigating action-relevant goal conflict often neglects the specific structure behind the conflicting goals, where goal conflict may be rooted. In addition, a broad conceptualization of goal conflict (see section When is a Goal Conflict a Goal Conflict? Answers From an Integrative Perspective on Goal Conflict) from a process-like perspective will probably entail presuppositions such as a person's ability for self-regulation as well as his or her ability to direct and regulate both cognition and emotion. Overall, a conceptualization of goal conflict based on an integrative perspective may clarify how goal conflicts emerge from multiple-goal pursuit.

In section Perspectives on Goal Conflicts, we have reviewed several ways in which a goal conflict may affect individuals' thoughts and actions. Each way in and of itself may be perceived by a person as no big deal, as a minor or major hassle, or as a catastrophe. For example, goal conflict that does not (yet) affect actions may be experienced quite differently—or not as negatively—compared to action-relevant goal conflict. Some individuals may be adversely affected by goal conflict at any stage due to their tendency to ruminate, whereas others simply do not worry about things that are not yet reality (cf. Brosschot and Thayer, 2004). Experiencing goal conflict with a goal one is hardly committed to may be resolved easily by disengaging from the goal, whereas a conflict between two highly valued goals may seriously impair a person's well-being. Thus, we would expect a complex interplay between the goal conflict and situational and personal characteristics that determine how individuals experience and react to goal conflict. Therefore, we need to make a greater effort to conceptualize (and measure) goal conflict independent of its immediate consequences on experience and behavior.

Conceptualizing personal goal conflict as set within multiple goal systems, of which every goal system encompasses multiple levels, leads to two immediate requirements for future research on personal goal conflict:

1. Consideration of levels of abstraction and action phases in the conceptualization of goal conflict and, thereby.
2. Consideration of interactions across levels of abstraction between goal systems and goal-pursuit (i.e., how goals in a multiple goal-pursuit context are broken down to means and how goal systems may conflict across levels).

### Methodological implications: three key decisions for empirical research on personal goal conflict

An integrative perspective should scrutinize the potential overlap of empirical approaches to the study of goal conflict favoring one perspective or the other, and attend to goal/mean conflict across levels of abstraction and in differing action phases. To handle this research objective, we suggest empirical studies combining both perspectives and related methodological approaches. This could be done in several ways, some of which we will outline in the following.

With respect to goal conflict as cognitive constructs, researchers should provide detailed instruction to help participants judge their goal interrelations. Maybe such instructions will use experiential instances to help participants identify goal conflict. With respect to experiential instances of goal conflict, a discussion of experiential indicators of goal conflict—for example, motivational interference (Fries et al., 2008; Grund et al., 2015), hesitation, or extensive rumination about one's goals—is needed to find a sound basis for empirical operationalization. On these grounds, empirical studies may reveal some overlap of the perspectives and may help find indicators of goal conflict, insight into moderators that affect individuals' perception and experience of goal conflict (e.g., goal properties, resource vs. inherent goal conflict), and ways to assess goal conflict.

To disentangle structural and procedural aspects of goal conflict, studies could combine goal conflict assessments based on cognitive constructs and experiential instances and, thereby, investigate interrelations of and reciprocal effects between goal conflict on different levels of abstraction and in different action phases, respectively. For example, participants may be asked to report cognitions about goal conflict and then track their actions and experience over the course of 1 or 2 weeks to report situations in which initially detected goal conflict actually impairs actions and—potentially—experiences. Further, after having experienced action-relevant goal conflict, participants should report on consequences for their current goal systems, for example, whether they have changed or disengaged from goals. A combination of quantitative and qualitative methodology appears apt to accommodate the complexity of goal conflict as well as possible.

Against this background and drawing on our review of the literature in section Methodological Approaches in Existing Research on Goal Conflict, three key decisions need to be seriously addressed, and interpretation of the results should be done in light of these key decisions and their related theoretical backgrounds.

1. The validity of assessing goal conflict as a cognitive construct and the choice of experiential instances indicating goal conflict need deliberate and more detailed methodological investigation.
2. An integrative perspective emphasizes that goal conflict occurs in goal interrelations. Hence, goal conflict should be assessed based on goal interrelations, whereas conceptualizations of conflict as attached to a single goal do not appear appropriate to reflect goal conflict.
3. The type of goal conflict needs to be made explicit in the instruction to ensure that participants report comparable goal conflicts.

Once we have clarified the definition and operationalization of goal conflict, we can move on to gain insight into how goal conflict affects individuals' actions and well-being and how individuals may best cope with goal conflict.

### Limitations of the present review

The present review focused personal goals, that is to say, goals set on a mid-level of abstraction that guide individuals' behavior over an extended period. We assume that personal goals are consciously accessible to individuals and, therefore, goal-pursuit as well as struggles to pursue and attain such goals may be verbalized. However, we acknowledge that unconscious processes of goal activation and goal-shielding are important to understand the extent and impact of goal conflict in people's everyday lives (Kleiman and Hassin, 2011). Hence, broadening the view to include subconscious processes of self-control and self-regulation, goal-like constructs that are not attached to personal goals but nevertheless affect people's course of action (e.g., desires, Hofmann et al., 2012) need to be taken into consideration as well (for an overview see Fishbach and Ferguson, 2007; Dijksterhuis and Aarts, 2010).

By focusing on goal conflict as a keyword, we excluded research that might be considered closely related to goal conflict, such as studies on work-life balance (e.g., Beauregard and Henry, 2009) or role conflict (e.g., Jackson and Schuler, 1985). Because goals may oftentimes arise from social roles (e.g., being a parent, being an employee), some role conflict may directly translate into goal conflict. Hence, these lines of research may offer important insights and should be considered in future research on goal conflict when the goal conflict under investigation encompasses several life domains or social roles, for example.

In addition, goal properties (Austin and Vancouver, 1996) may play a vital role in the experience of and reaction to goal conflict. In this sense, the notion of vertical goal conflict or self-concordance of subordinate goals in relation to higher-order self-defining goals and needs within one goal system (cf. Sheldon and Kasser, 1995) seems crucial also with regard to conflicts between two or more goal systems (Senécal et al., 2001; Gorges et al., 2014).

### Concluding remarks

In sum, goal conflict is a highly complex construct that needs more careful conceptualization and definition prior to empirical investigations and consistent transformations of these definitions into ways to measure goal conflict. Within our review of theoretical and empirical approaches to the study of goal conflict, we have summarized and highlighted potential paths that may be taken to further our understanding of goal conflict. Learning more about the complex interplay of multiple factors that precede goal conflict and lead to its detrimental effects will enable us to see threats of (rising) goal conflict quite early and probably help people to minimize interruptions by, or even avoid, goal conflict.

Our intention was to help arrange ideas and to increase awareness with respect to the many details that need attention in studies on goal conflict. We would like to invite researchers to tackle the challenges outlined in this article. We should make an effort to develop comparable research designs to promote converging evidence and to arrive at a common ground regarding the conceptualization of goal conflict. It may be wise to distinguish between a narrow definition, a broad definition, and maybe even a definition in between to get hold of goal conflict from different theoretical angles. In fact, we refer to goal conflict as a moving target in the title of this manuscript because in writing this review, goal conflict kept changing its form, theoretical foundations, and hypothetical effects in our minds. Because conceptualizations of goal conflict heavily influence empirical approaches to the study of goal conflict and—consequently—interpretation of empirical findings, we need to be more attentive to conceptual details and more explicit about presuppositions when doing research on goal conflict. A more integrated approach and consideration of related lines of research should hold great potential for the critical advancement of theory building and empirical research with respect to antecedents, experience, and consequences of goal conflict. Its complexity and sensitivity to different perspectives will make research on goal conflict difficult. Nevertheless, we hope that intraindividual personal goal conflict will be addressed more in the future and that our account of goal conflict presented here will be of relevance to these studies and their results, in an attempt to further our conceptualization and understanding of goal conflict.

## Author contributions

JG was the leading author of this manuscript; AG contributed as a co-author. JG developed the outline and wrote the introduction, the section on the integrative perspective, the systematic literature review, and the conclusions. AG contributed the theoretical background on the structure-like and the process-like perspective and discussed all other sections with JG. Both authors read and approved the manuscript for publication.

### Conflict of interest statement

The authors declare that the research was conducted in the absence of any commercial or financial relationships that could be construed as a potential conflict of interest.
